# Effect of high-flow high-volume-intermittent hemodiafiltration on metformin-associated lactic acidosis with circulatory failure: a case report

**DOI:** 10.1186/s13256-018-1809-6

**Published:** 2018-09-29

**Authors:** Kodai Suzuki, Hideshi Okada, Shozo Yoshida, Haruka Okamoto, Akio Suzuki, Keiko Suzuki, Yuto Yamada, Hideki Hayashi, Ryu Yasuda, Tetsuya Fukuta, Yuichiro Kitagawa, Takahito Miyake, Tomonori Kawaguchi, Takatomo Watanabe, Tomoaki Doi, Keisuke Kumada, Hiroaki Ushikoshi, Tadashi Sugiyama, Yoshinori Itoh, Shinji Ogura

**Affiliations:** 1 Department of Emergency and Disaster Medicine, Gifu University Graduate School of Medicine, 1-1 Yanagido, Gifu, 501-1194 Japan; 2 Department of Pharmacy, Gifu University Hospital, Gifu, Japan; 3 Laboratory of Pharmacy Practice and Social Science, Gifu Pharmaceutical University, Gifu, Japan; 4 Community Healthcare Pharmacy, Gifu Pharmaceutical University, Gifu, Japan; 5 Division of Clinical Laboratory, Gifu University Hospital, Gifu, Japan

**Keywords:** Metformin, Metformin-associated lactic acidosis, Hemodiafiltration, Diabetes

## Abstract

**Background:**

Metformin-associated lactic acidosis is a well-known life-threatening complication of metformin. We here report the case of a patient who developed metformin-associated lactic acidosis without organ manifestations, due to the simultaneous ingestion of an overdose of metformin and alcohol, and who recovered with high-flow high-volume intermittent hemodiafiltration.

**Case presentation:**

A 44-year-old Asian woman with type 2 diabetes attempted suicide by ingesting 10 tablets of metformin 500 mg and drinking approximately 600 mL of Japanese sake containing 15% alcohol. She was transferred to our emergency department because of disturbed consciousness. Continuous intravenous administration of noradrenalin (0.13 μg/kg per minute) was given because she was in shock. Laboratory findings included a lactate level of 119 mg/dL (13.2 mmol/L), bicarbonate of 14.5 mmol/L, and serum metformin concentration of 1138 ng/mL. She was diagnosed as having metformin-associated lactic acidosis worsened by alcohol. After 4560 mL of bicarbonate ringer (Na^+^ 135 mEq/L, K^+^ 4 mEq/L, Cl^−^ 113 mEq/L, HCO_3_^−^ 25 mEq/L) was administered, high-flow high-volume intermittent hemodiafiltration.

(dialysate flow rate: 500 mL/min, substitution flow rate: 3.6 L/h) was carried out for 6 h to treat metabolic acidosis and remove lactic acid and metformin. Consequently, serum metformin concentration decreased to 136 ng/mL and noradrenalin administration became unnecessary to maintain normal vital signs. On hospital day 12, she was moved to the psychiatry ward.

**Conclusions:**

HFHV-iHDF may be able to remove metformin and lactic acid efficiently and may improve the condition of hemodynamically unstable patients with metformin-associated lactic acidosis.

## Background

Currently, metformin is the first-line agent for the treatment for type 2 diabetes [1]. Metformin-associated lactic acidosis (MALA) is a well-known, life-threatening, rare complication, with an incidence of less than 10 per 100,000 patients a year [2]. Intensive care management is known to have gradually decreased the MALA-associated mortality rate. However, the mortality rate is still more than 20% [3].

MALA usually occurs in metformin-treated diabetic patients with kidney manifestations due to reduced metformin excretion. Additionally, renal insufficiency decreases hydrogen excretion by the kidneys and leads to the development of acidosis independent of metformin [4]. Even if kidney function is normal, metformin overdose or ingestion of alcohol are risk factors for developing MALA. In kidney or non-kidney comorbidity, treatment for MALA comprises conventional renal replacement therapy (RRT), particularly continuous venovenous hemofiltration (CVVH) or hemodialysis (HD); however, the effort of RRT itself has been still unknown in the treatment for MALA due to both metformin overdose and alcohol ingestion.

We here report the case of a patient who developed MALA without organ manifestations by ingesting an overdose of metformin and alcohol simultaneously and who recovered with high-flow high-volume intermittent hemodiafiltration (HFHV-iHDF). This case suggests that HFHV-iHDF may become effective therapy for hemodynamically unstable patients with MALA due to simultaneous alcohol ingestion.

## Case presentation

A 44-year-old Asian woman with type 2 diabetes and schizophrenia was being treated at our hospital. For type 2 diabetes, she received 1250 mg of metformin and 50 mg of sitagliptin phosphate hydrate a day and an intermediate-acting insulin 6 unit injection before bedtime. For schizophrenia, she received 8 mg of biperiden hydrochloride, 600 mg of valproic acid, 600 mg of carbamazepine, 15 mg of mirtazapine, 25 mg of clomipramine, 25 mg of chlorpromazine, 12.5 mg of promethazine, and 40 mg of phenobarbital. There was no other medical history. Her father had type 2 diabetes. She smokes two packs of cigarettes per day for 24 years, and drinks socially. After a quarrel with her mother, she attempted suicide by ingesting 10 tablets of 500 mg metformin and drinking about 600 mL of Japanese sake containing 15% alcohol.

She was transferred to our emergency department because of disturbed consciousness. On physical and neurological examination, her Glasgow Coma Scale was 3 (eye, 1; verbal, 1; motor, 1). Both pupils were 1.5 mm, and light reflexes were rapid. Her respiratory rate was 30 breaths per minute. Her heart rate was 120 beats per minute and blood pressure was 120/60 mmHg. She then received continuous intravenous administration of noradrenalin (0.13 μg/kg per minute) because she was in shock. Her body temperature was 35.5 °C. On auscultation, no crackles and wheezing were detected. There were no murmurs. Aspiration pneumonia was detected in both the lungs by computed tomography. Laboratory findings (normal ranges in parentheses) demonstrated aspartate transaminase level of 16 (7 to 35) IU/L, alanine transaminase level of 15 (7 to 40) IU/L, lactate dehydrogenase level of 273 (125 to 225) IU/L, creatinine level of 0.94 (0.60 to 1.20) mg/dL, and blood urea nitrogen level of 12.7 (8 to 20) mg/dL. Arterial blood gas analysis on 100% oxygen revealed a pH of 7.067, partial pressure of oxygen in arterial blood of 143 mmHg, partial pressure of carbon dioxide in arterial blood of 52.9 mmHg, bicarbonate of 14.5 mmol/L, base excess of − 16 mmol/L, lactate of 119 mg/dL (13.2 mmol/L), and blood glucose of 632 mg/dL. Serum metformin concentration was 1138 ng/mL (Table 1). Serum osmolality and sodium concentration were 309 mOsm/kg and 135 mEq/L, respectively. No ketone bodies were detected in her urine.

**Table 1:** Laboratory findings on admission

Biochemistry	
Total protein (g/dL)	5.7
Albumin (g/dL)	3.6
Aspartate transaminase (IU/L)	16
Alanine transaminase (IU/L)	15
Lactate dehydrogenase (IU/L)	273
Alkaline phosphatase (IU/L)	225
γ-glutamyltranspeptidase (IU/L)	35
Creatinine (mg/dL)	0.94
Blood urea nitrogen (mg/dL)	12.7
Sodium (mEq/L)	135
Potassium (mEq/L)	3
Chloride (mEq/L)	96
Total bilirubin (mg/dL)	0.2
C-reactive protein (mg/dL)	0.39
Hemoglobin A1c (%)	9.9
Blood sugar (mg/dL)	632
Serum osmolality (mOsm/kg)	135
Metformin concentration (ng/mL)	1138
Complete blood cell counts	
White blood cell (/μL)	25,320
Red blood cell (× 10^4^/μL)	459
Hemoglobin (g/dL)	13.7
Platelet (×10^4^/μL)	24.9
Coagulation status	
Activated partial thromboplastin time (sec)	30.3
Prothrombin time (%)	> 120
Prothrombin time-international normalized ratio	0.86
Arterial blood gas	
F_I_O_2_	1.0
pH	7.067
PaCO_2_ (mmHg)	52.9
PaO_2_ (mmHg)	143
HCO_3_- (mmol/L)	14.5
Base excess (mmol/L)	−16
Lactate (mg/dL)	119
(mmol/L)	13.2

**Fig. 1:**
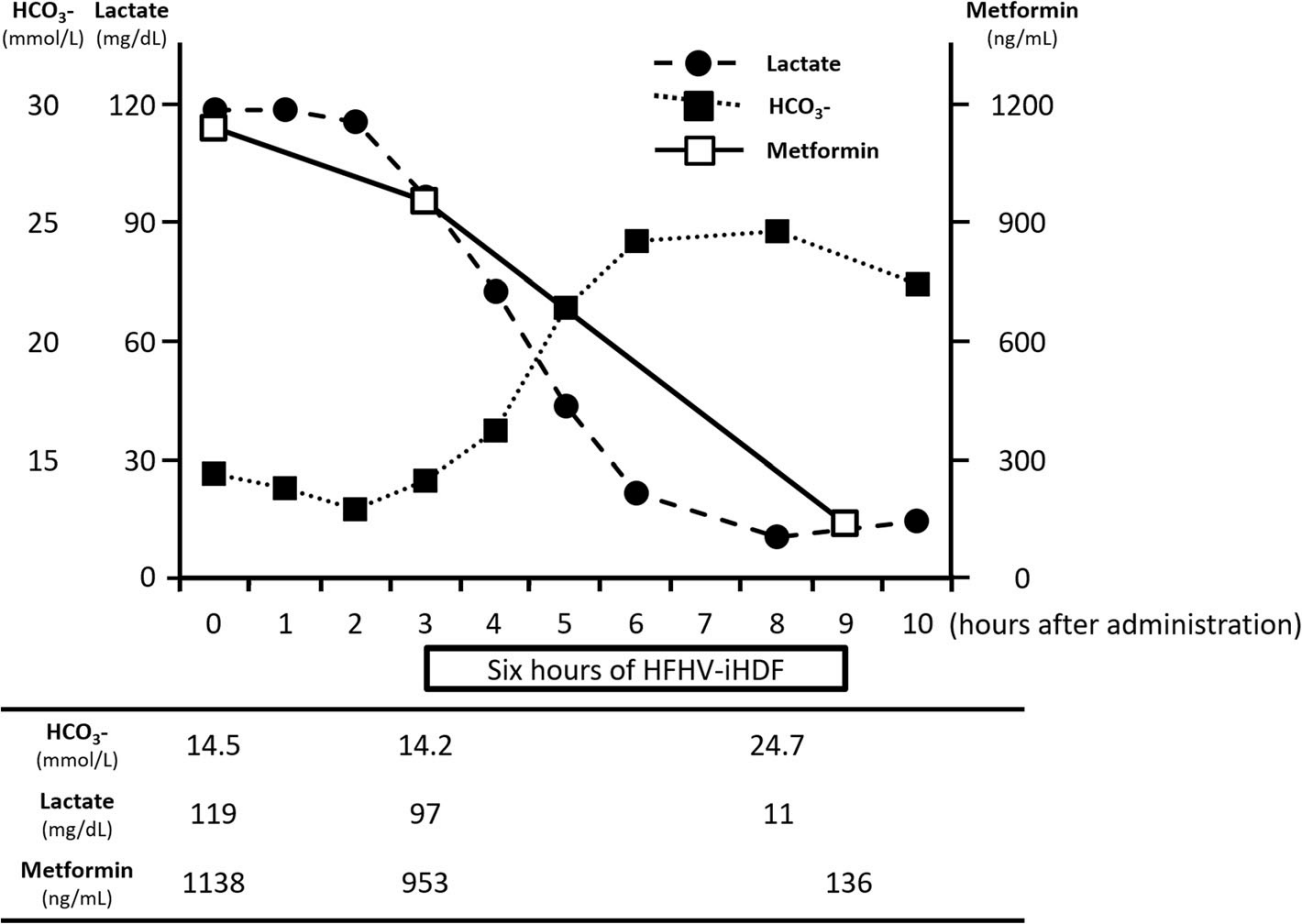
Plot of bicarbonate (*black rectangle*), lactate (*filled circle*), and metformin (*rectangle*) versus time. *HCO*_*3*_*-* bicarbonate, *HFHV-iHDF* high-flow high-volume intermittent hemodiafiltration

She was diagnosed as having MALA worsened by alcohol. First, enforced sedation with midazolam was performed because of restlessness, tracheal intubation was carried out, and activated carbon and magnesium citrate were used for decontamination after gastric lavage. After 4560 ml of bicarbonate ringer (Na^+^ 135 mEq/L, K^+^ 4 mEq/L, Cl^−^ 113 mEq/L, HCO_3_^−^ 25 mEq/L) was administered, HFHV-iHDF (dialysate flow rate: 500 mL/min, substitution flow rate: 3.6 L/h) was carried out for 6 h using a polysulfone high-performance membrane (APS-15E, Asahi Kasei Medical, Tokyo, Japan) to treat metabolic acidosis and remove lactic acid and metformin. After that, her serum metformin concentration decreased to 136 ng/mL (Fig. 1) and noradrenalin administration became unnecessary to maintain normal vital signs. On hospital day 2, HFHV-iHDF was carried out again, which improved her consciousness. On hospital day 10, she was extubated after aspiration pneumonia healed. On hospital day 12, she was moved to the psychiatry ward. She left the hospital 1 month after she moved to the psychiatry ward. When she left the hospital, she was discontinued from metformin administration. Although there is no recurrence of lactic acidosis, she was re-hospitalized into the psychiatry ward because she took an overdose of sleeping pills, 2 months after she left the hospital. Since then, she was in the hospital for the control of her schizophrenia and diabetes for more than 2 months.

## Discussion and conclusions

In the present case, the lactic acidosis was caused by an excessive consumption of alcohol and metformin, in spite of no renal dysfunction. CVVH was generally performed for cases like this [5]. However, HFHV-iHDF was performed under stabilization of hemodynamics in the present case [5], and it was successful for rapidly removing metformin and lactic acid.

Metformin, a first-line drug for type 2 diabetes, has a molecular weight of 165 g/mol, high water solubility, minimal plasma protein binding, and a large volume of distribution [4, 5]. It is excreted predominantly unchanged by the kidneys [4]. RRT is considered adequate for the treatment of MALA because it efficiently removes metformin and lactic acid by diffusion through the dialyzer membrane [6]. Additionally, a bicarbonate-buffered and high-sodium dialysate improves the metabolic acidosis and increases blood volume, thus enhancing the renal blood flow [6].

CVVH and HD are some of the RRT methods that were previously studied. The clearance of a drug by CVVH was less than that with conventional HD. Therefore, CVVH should only be considered in patients who are too hemodynamically unstable to tolerate HD [7]. The reason of selecting HFHV-iHDF therapy for this hemodynamically unstable patient was that this therapy could remove metformin and lactate molecules more efficiently and sooner than CVVH does. A previous study reported that the plasma concentration of metformin after 24 h of CVVH was about one-third of that before CVVH. [5] Our data suggested that the plasma concentration of metformin after 6 h of HFHV-iHDF was about one-seventh as that before HFHV-iHDF. Considering MALA pathogenesis, removing metformin is a fundamental solution for curing MALA. A recent study suggested that metformin and lactate plasma concentration significantly correlated with mortality [8]. In HFHV-iHDF, hemodynamics of patients with critical MALA could worsen because of a large dialysate flow rate and a high filtration flow rate. However, HFHV-iHDF can be conducted for patients with MALA if sufficient transfusion can be maintained and under adequate critical care management.

MALA can occur in patients without organ manifestations due to the simultaneous ingestion of an overdose of metformin and alcohol. Several factors are involved in the development of MALA because metformin has multiple mechanisms of producing lactic acidosis. Simultaneous ingestion of alcohol simultaneously markedly promotes the development of MALA. Metformin decreases blood sugar concentration by reducing gluconeogenesis, increasing the peripheral uptake of glucose, and decreasing fatty acid oxidation [4]. The inhibition of gluconeogenesis by metformin is localized to the primary energy-consuming reactions, and in the negative shift of the NADH/NAD+ redox potential [9]. Alcohol ingestion causes a sharp rise in hepatic NADH/NAD+ ratio, which in turn inhibits gluconeogenesis from lactate [9]. The MALA in our patient was caused by ingestion of an overdose of metformin (5 g); however, this dose was considered too small to cause MALA [10]. A report suggested that increasing the dose was associated with the development of MALA, with a median ingested dose of 15 g causing lactic acidosis compared to 2.8 g without lactic acidosis [10]. In this case, removal of metformin by HFHV-iHDF was useful, because alcohol ingestion influenced metformin metabolism.

This case demonstrated the effectiveness of HFHV-iHDF for MALA due to simultaneous alcohol ingestion. HFHV-iHDF may improve the conditions of hemodynamically unstable patients with MALA.
